# Lysosomal channel TPC2 modulates microglia-endothelial signaling in choroidal angiogenesis

**DOI:** 10.1007/s10456-026-10082-4

**Published:** 2026-08-12

**Authors:** Yi Lu, Alice Reschigna, Franz Kynast, Zhuo Yang, Maximillian Gerhardt, Pavel Kielkowski, Siegfried Priglinger, Martin Biel, Stylianos Michalakis

**Affiliations:** 1 Department of Ophthalmology, LMU University Hospital, LMU Medizin, LMU Munich, Munich, Germany; 2 Department of Chemistry, Ludwig-Maximilians-Universität München, Munich, Germany; 3 Department of Pharmacy, Ludwig-Maximilians-Universität München, Munich, Germany; 4 Munich Center for Cell & Gene Therapy, LMU University Hospital, LMU Medizin, LMU Munich, Munich, Germany

**Keywords:** TPC2, Choroidal neovascularization, Lysosome, Microglia, Cathepsins

## Abstract

Pathological choroidal neovascularization underlies vision loss in neovascular age-related macular degeneration (nAMD), yet the molecular regulators coordinating vascular and immune components remain incompletely defined. Here, we investigated the role of the endolysosomal cation channel, two-pore channel 2 (TPC2) in choroidal angiogenesis. Loss of TPC2 in mice markedly reduced ex vivo choroidal sprouting, while pharmacological activation enhanced vascular growth. Mechanistically, *Tpc2*-deficiency led to downregulation of multiple microglia-derived pro-angiogenic factors and impaired the ability of the microglial secretome to stimulate neovascularization. In choroidal vascular cells, TPC2 loss attenuated NF-κB/MAPK signaling pathways. *Tpc2*-deficiency is also associated with lysosomal secretion of cathepsins, especially CTSD, resulting in decreased extracellular proteolytic activity and impaired paracrine regulation of angiogenesis. Extending these findings to human cells, *TPC2* knockout in iPSC-derived endothelial cells impaired migration, tube formation, and CTSD activity in the secretome, mirroring the murine phenotype. Together, these results establish TPC2 as one of the regulators of lysosome-mediated choroidal angiogenesis, highlighting its potential as a therapeutic target in nAMD.

**Supplementary Information:**

The online version contains supplementary material available at 10.1007/s10456-026-10082-4.

## Introduction

Age-related macular degeneration (AMD) is a leading cause of irreversible vision loss worldwide, particularly in the elderly population [1]. It is classified into two major forms: dry (atrophic) AMD, characterized by the accumulation of drusen and progressive retinal pigment epithelium (RPE) degeneration, and wet (neovascular) AMD (nAMD), which involves the pathological formation of choroidal neovascularization (CNV) [2]. CNV leads to the growth of abnormal blood vessels from the choroid through Bruch’s membrane into the subretinal space, resulting in exudation, hemorrhage, and fibrosis, which ultimately contribute to severe vision loss [3]. Current treatment strategies primarily rely on anti-vascular endothelial growth factor (VEGF) therapy, which has significantly improved visual outcomes for many patients. However, its limitations, including incomplete response, potential development of geographic atrophy, and long-term progression to fibrosis, highlight the need for alternative therapeutic approaches [4, 5].

Both pathological angiogenesis and inflammation play critical roles in CNV development [6, 7]. Choroidal endothelial cells actively proliferate and invade the subretinal space in response to angiogenic factors such as VEGF, fibroblast growth factors (FGFs), and platelet-derived growth factors (PDGFs) [8]. In parallel, inflammation contributes to disease progression by modulating the CNV microenvironment. Retinal microglia, the tissue-resident immune cells of the retina, are key regulators of this process. Under physiological conditions, microglia maintain homeostasis by removing cellular debris and secreting neurotrophic factors [9]. However, during CNV, they become activated and accumulate in the subretinal space, where they secrete cytokines, chemokines, and matrix metalloproteinases (MMPs), facilitating extracellular matrix (ECM) remodeling and neovascularization [10]. Despite extensive research, the molecular mechanisms that regulate microglial activation and their crosstalk with choroidal endothelial cells remain poorly understood.

Recent studies suggest that two-pore channel 2 (TPC2), a lysosomal cation channel, may play a crucial role in both pathological angiogenesis and inflammation [11, 12]. TPC2 belongs to the two-pore channel family, which consists of TPC1 and TPC2, both of which are primarily localized to endolysosomal membranes [13]. TPC2 is activated by second messengers such as nicotinic acid adenine dinucleotide phosphate (NAADP) and phosphatidylinositol 3,5-bisphosphate (PI(3,5) P₂), enabling it to mediate calcium (Ca²⁺) and sodium (Na⁺) fluxes across the lysosomal membrane [14]. Through its role in lysosomal trafficking, calcium signaling, and vesicular secretion, TPC2 is involved in a variety of cellular processes, including autophagy [15] and exocytosis [16]. These functions are fundamental to endothelial cell migration, interaction with ECM, and angiogenic potential. Additionally, TPC2 has been implicated in retinal microglia inflammatory responses, particularly in cell activation and cytokine secretion [17]. Given the dual involvement of TPC2 in both angiogenesis and inflammation, its role in nAMD requires further investigation. In this study, we reveal the role of TPC2 in nAMD by investigating its effects on retinal microglia, choroidal vascular cells, and their interaction. By elucidating these mechanisms, we aim to provide new insights into the lysosomal regulation of inflammation and neovascularization, potentially identifying TPC2 as a novel therapeutic target for nAMD and other neovascular diseases.

## Materials and methods

### Mice

WT C57BL/6J and *Tpc2*^*−/−*^ animals were raised in 12-hour (h) light-dark cycles with food (Ssniff; regular feed: R/M-H; breeding feed: M-Z Extrudat) and water. *Tpc2*^⁻/⁻^ mice were originally generated as describe [18] and were backcrossed onto a C57BL/6J background for more than 10 generations. WT mice are derived from subsequent heterozygous breedings within the same colony, and they are frequently refreshed.

### Choroidal sprouting assay

Choroidal sprouting assay was carried out as previously described [19] with slight modifications. Briefly, pieces of the RPE/choroid/sclera complex were dissected from peripheral region of eyes of post-natal ages (P)25–35 mice, cut into 1 × 1-mm fragments and then embedded in growth factor-reduced Matrigel (Cat.354230, Corning, USA) in 24-well plates. The Matrigel was allowed to solidify for approximately 20 min (min) at 37 °C before adding culture medium. Then the explants were cultured in EBM-2 complete medium consisting of EBM-2 Basal Medium (Cat. CC-3156, Lonza, Switzerland) supplemented with the Microvascular Endothelial SingleQuots kit (Cat. CC-4177, Lonza, Switzerland), which contains 5% fetal bovine serum (FBS), VEGF (50 ng/mL), hFGF-B (10 ng/mL), R3-IGF-1 (50 ng/mL), hEGF (50 ng/mL), hydrocortisone (0.4 µM), ascorbic acid (50 µg/mL), heparin (10 µg/mL), and 1%GA-1000 (Antibiotic-Antimycotic). Culture was maintained at 37 °C with 5% CO_2_ for 6 days. In pharmacological experiments, tissue pieces were cultured for 4 h in full medium once embedded in Matrigel. Thereafter, medium was replaced with fresh medium containing 10 µM SG-094, 5 µM TPC2-A1P (kindly provided by Prof. Franz Bracher) or 200 ng/mL CHIL3 (Cat. HY-P7845, MCE, USA). Images of tissue pieces were taken with EVOS M5000 microscope (Thermo Fisher Scientific, USA) on specific days. Sprouting areas were analyzed using the ImageJ software (National Institutes of Health, USA) with SWIFT-choroid macros developed by Z Shao and M Friedlander [19].

### Cell line culture

BV2 murine microglial cell line (Accegen Biotechnology, USA) and RAW264.7(ATCC, USA) murine macrophage cell line were maintained in DMEM+GlutaMAX (Cat. 31966021, Gibco) supplemented with 10% FBS (Thermo Fisher Scientific, USA) and 1% penicillin/streptomycin (P/S; Gibco). Cells were cultured at 37 °C in a humidified incubator with 5% CO₂ and passaged upon reaching approximately 80–90% confluence. Culture medium was replaced every 2–3 days.

### Primary choroidal vascular cell culture

Primary choroidal vascular cells (pCVCs) were isolated from sprouting choroid explant cultures as described above. 200 µL 0.25% trypsin-EDTA (Cat. 25200056, Gibco, USA) was added to each well after 7–10 days of culturing. After 5-min incubation at 37 °C, 300 µL pre-warmed EBM-2 complete medium was added and the tissue/Matrigel complex was pipetted up and down vigorously to create single-cell suspension before passing the solution through a 100 μm cell strainer (Cat. 732–2759, VWR, USA). The cell-strainer was then rinsed with media, and the cells were centrifuged for 5 min at 400×*g*. The media was aspirated, and the cell pellet resuspended again and transferred into an appropriate culture flask. Cells were cultivated in EBM-2 complete medium as described above. All experiments were performed using passage 1 (P1) cells.

### Primary retinal microglia culture

Primary retinal microglia-enriched (pRMG) culture were isolated from (P)25–35 WT and *Tpc2*^⁻/⁻^ mice using a papain-based enzymatic dissociation protocol. Typically, retinas from 4 to 5 mice were pooled to obtain enough microglia cells. Mice were sacrificed, and eyes were enucleated immediately. Under a stereomicroscope, retinas were carefully dissected and enzymatically digested for 15 min at 37 °C according to Feodorova et al. [20] with minor modifications. The suspension was filtered through a 40-µm cell strainer and plated into an appropriate culture flasks. The culture medium was based on DMEM-Ham’s F-12 (Thermo Fisher Scientific, USA), 10% FBS, 0.45% D-(+)-glucose (Sigma-Aldrich, USA), 1.5 µg/mL ovine wool cholesterol (Sigma Aldrich, USA), 1× GlutaMAX (Thermo Fisher Scientific, USA), 1 ng/mL murine granulocyte-macrophage colony-stimulating factor (GM-CSF; PeproTech, USA), and 1× P/S. Mixed glial cultures were maintained at 37 °C in a humidified 5% CO₂ incubator, with half of the medium replaced every 3–4 days. After 4–6 weeks in vitro, the cultures were enriched for retinal microglia. Adherent astrocytes/Müller glia were removed by incubation for 5 min at 37 °C with 0.08% Trypsin-EDTA in Phosphate-buffered saline (PBS), exposing microglia cells firmly attached to the bottom of the flask. Afterwards pRMG were collected by scraping with a cell scraper. After centrifugation for 5 min at 300×*g*, cells were carefully resuspended in microglia medium, counted and seeded at a density of 80,000–100,000 cells/well in a 24-well. For subsequent immunostaining, glass coverslips coated with 20 µg/mL Poly-D-Lysine (PDL; Sigma-Aldrich, USA) in PBS were placed in the wells prior to seeding.

### Flow cytometry analysis

Primary mouse choroidal vascular cells were detached using 0.25% trypsin-EDTA at 37 °C for 5 min. The resulting cell suspension was passed through a 100-µm cell strainer and centrifuged at 400×*g* for 5 min to collect the choroidal vascular cells. Endothelial cells were identified by staining with phycoerythrin (PE)-conjugated anti-Endomucin antibody (1:100, Cat. 2647665, Invitrogen, USA) and allophycocyanin (APC)-conjugated anti-CD31 antibody (1:100, Cat. 551262, Invitrogen, USA). Adherent retinal microglia were detached from the culture plate using a cell scraper and centrifuged at 300×*g* for 5 min. Microglia were then labeled with Phycoerythrin–Cyanine7 (PE-Cy7)-conjugated anti-CD45 antibody (1:100, Cat. 2629030, Invitrogen, USA) and APC-conjugated anti-CD11b antibody (1:100, Cat. 2629033, Invitrogen, USA). After 1 h of staining at 4 °C in the dark, cells were washed three times with FACS buffer (2% FBS and 2 mM EDTA in PBS), each washing lasting 10 min. Cell viability was negatively gated on DAPI. Fluorescence intensity was acquired using a flow cytometer (LSRFortessa, BD Biosciences, USA), and data were analyzed with FlowJo software (BD Biosciences, USA).

### Tube formation assay

The angiogenic potential of pCVCs and human iPSC-derived endothelial cells was assessed using a tube formation assay on Matrigel. µ-Slide 15 Well angiogenesis chambers (ibidi, Germany) were pre-coated with 10 µL of growth factor-reduced Matrigel per well. The slides were incubated at 37 °C for 20 min. In this way, Matrigel polymerizes and forms a gel-like matrix. Cells were harvested and resuspended in endothelial growth medium at a concentration of 2 × 10⁵ cells/mL. A total of 10,000 cells in 50 µL of EBM-2 complete medium were seeded into each Matrigel-coated well. The slides were then incubated in a humidified CO₂ incubator at 37 °C to allow tube-like structures to form. At the end of the incubation period, images were captured for each well using an EVOS M5000 microscope. Quantitative analysis of tube formation was performed using the Angiogenesis Analyzer plugin [21] in ImageJ software.

### Cell migration assay

iPSC-induced endothelial cells (iEC) (WT and *TPC2*^*−/−*^, see detailed generation and differentiation below) were resuspended in complete EGM-2 medium (Promo Cell, Germany) at a final concentration of 5 × 10⁵ cells/mL. A volume of 70 µL of the cell suspension was carefully pipetted into each well of a two-well culture insert (ibidi, Germany) placed in a 6-well plate. Cells were allowed to attach and grow for 4 h at 37 °C in a humidified incubator with 5% CO₂. After this incubation period, the insert was gently removed using sterile forceps, creating a defined, reproducible cell-free gap of approximately 500 μm between the two cell monolayers. The wells were then washed once with pre-warmed PBS to remove any non-adherent cells and replaced with fresh EGM-2 complete medium. Images of the cell-free area were acquired immediately after insert removal (0 h) and at defined time points. The width of the remaining cell-free gap was measured using Cell watcher M (Phio, Germany), and gap closure was quantified by calculating the percentage of the original wound area covered by migrating cells.

### Sample preparation for mass spectrometry (MS)

Equal numbers of cells (pRMG or pCVCs from WT and *Tpc2*^⁻/⁻^) were seeded and cultured. Cell viability was monitored throughout the experiments and no substantial differences were observed between groups at the time of sample collection.

For cell lysates, cells were harvested by scraping in cold PBS and centrifuged at 300–400×*g* for 5 min at 4 °C. The cell pellets were lysed in RIPA buffer (Thermo Fisher Scientific, USA) supplemented with a protease and phosphatase inhibitor cocktail (Roche, USA) on ice for 30 min. Lysates were then centrifuged at 14,000×*g* for 15 min at 4 °C to remove insoluble debris.

For secretome samples, supernatants were collected and centrifuged at 300–400×*g* for 5 min to remove floating cells, followed by a second centrifugation at 2000×*g* for 10 min to eliminate cellular debris. The cleared supernatant was then precipitated by adding four volumes of cold acetone and incubating at − 20 °C for overnight to allow protein precipitation. The supernatant samples were centrifuged at 10,000×*g* for 10 min at 4 °C to pellet the proteins. The protein pellet was washed twice with cold acetone and after drying, the pellet was resuspended in RIPA buffer for further processing.

The protein concentration of cell lysates and supernatant was determined using the BCA protein assay kit (Thermo Fisher Scientific, USA). Protein samples were prepared and digested using the SP3 protocol with paramagnetic beads as previously described [22]. 20 µL carboxylate-coated magnetic beads (1:1 mixture of hydrophilic and hydrophobic beads) were washed manually thrice with 100 µL MS-scale water and the last washing solution was kept within the 96-well plate. Equal amounts of total protein (20 µg) were diluted to 50 µL with PBS buffer and added onto the beads. The mixture was shaken for 1 min at 850 rpm, room temperature (RT). Afterwards 60 µL absolute ethanol was added to each sample and incubated for 5 min at 850 rpm, RT. The supernatants were removed, and the protein-bound beads were washed three times with 100 µL 80% ethanol and once with 100 µL acetonitrile, each followed by a 1-min incubation at 850 rpm at RT. After the final wash, beads were resuspended in 100 µL 100 mM ammonium bicarbonate and digested overnight with 1 µL sequencing-grade trypsin (0.5 mg/20 mL, Promega, USA) at 37 °C, 600 rpm. On the next day, the peptide mixtures were all transferred to new Eppendorf tubes, and the beads were washed with 50 µL and 30 µL 1% formic acid (FA) in water. The beads with washing solutions were incubated at 40 °C, 850 rpm for 5 min. The washing solutions were collected all together with digested peptide mixtures and placed within a MS-vial. For LC-MS/MS measurement, 5 µL solutions were injected for each sample.

### LC-MS/MS measurement and data analysis

Peptides were analyzed on an Orbitrap Eclipse Tribrid Mass Spectrometer coupled to an UltiMate 3000 Nano-HPLC system with nanospray ionization and FAIMS. Peptides were loaded onto a C18 precolumn and separated on an in-house packed C18 analytical column using a gradient of water and acetonitrile, both containing 0.1% FA. The mass spectrometer was operated in data-independent acquisition (DIA) mode [23].

Raw files were converted to mzML format using ProteoWizard and analyzed with DIA-NN 1.8.1. Peptides were searched against the Uniprot murine database including contaminants and decoys. The dataset quality metrics including DIA-NN output statistics were assessed and all identified proteins had Q-values below 0.01. Protein intensities were log2-transformed, and only proteins with at least two valid measurements out of three biological replicates were retained. Missing values imputed from normal distribution with 1.8 downwards shift, and differential expression was analyzed using a Student’s t-test with FDR correction. For Venn diagram analysis, protein presence was defined at the group level: a protein was considered detected if identified in at least two out of three biological replicates.

### Quantitative reverse transcription PCR (qRT-PCR)

qRT-PCR was performed on an Applied Biosystems QuantStudio 5 system using SYBR Green chemistry for detection. Total RNA was isolated from both choroidal vascular cells and retinal microglia using the Qiagen RNeasy kit (Qiagen, USA), following the manufacturer’s protocol for RNA extraction. For cDNA synthesis, 100 ng of total RNA from each sample was reverse transcribed using the Revert Aid First Strand cDNA Synthesis Kit (Thermo Fisher Scientific, USA). The reverse transcription reaction was carried out according to the manufacturer’s instructions to ensure efficient synthesis of complementary DNA. Gene expression levels were quantified using the 2^−ΔCt^ method, with *Gapdh* as the internal reference gene for normalization. Specific primers for the target genes were used. The relative expression of target genes was calculated by 2^−ΔΔCt^ method. Data analysis was performed using QuantStudio Design & Analysis Software (Thermo Fisher Scientific, USA).

### Immunofluorescence staining

Cells were fixed in 4% paraformaldehyde (PFA) for 15 min at RT, followed by permeabilization with 0.3% Triton X-100 for 10 min. Blocking was performed with 5% Chemiblocker for 1 h at RT to minimize non-specific binding. For choroidal vascular cells, primary antibodies were used against Endomucin (1:200, Cat. 4052388, Millipore, USA), and TPC2 (1:250, Cat. Ab119915, Abcam, USA). For retinal microglia, primary antibodies included CHIL3 (1:1000, Cat. PA5-81356, Invitrogen, USA), CD11b (1:500, Cat. B261558, BioLegend, USA), and TPC2. Primary antibody incubation was carried out overnight at 4 °C. Following primary antibody incubation, cells were incubated with secondary antibodies Alexa Fluor 488/647 (1:800, Cat. A-21121/A-21247, Invitrogen, USA) for 1 h at RT in the dark. Nuclei were counterstained with DAPI (1:1000) to visualize the cell nuclei. After staining, coverslips were mounted onto slides using an antifade medium to preserve fluorescence. Images were captured using Leica SP8 confocal microscope (Leica Microsystems, Germany).

### Western blot

Proteins from cell pellets and supernatant were extracted and quantified as described in MS preparation section. Equal amount of protein was separated by SDS-PAGE on a 10% polyacrylamide gel and transferred to PVDF membranes at 100 V for 1 h at 4 °C. Membranes were blocked with 5% non-fat milk in Tris-buffered saline with Tween 20 (TBST) for 1 h at RT, then incubated overnight at 4 °C with primary antibodies, including anti-CHIL3 (1:1000, Cat. PA5-81356, Invitrogen, USA). After 3 times of washing with TBST, membranes were incubated with HRP-conjugated anti-Rabbit IgG secondary antibodies (1:2000, Cat. 7074, CST, USA) for 1 h at RT. After 3 times of washing, signals were developed using ECL reagents (Bio-Rad, USA). Chemiluminescence was captured using an imaging system (Fusion Solo S, Vilber Lourmat).

### Cathepsin D activity assay

Choroid/RPE tissue, supernatant from choroidal sprouting assays, from primary choroidal vascular cell cultures and from iEC were processed for Cathepsin D (CTSD) activity measurement. For supernatant samples, cells or explants were washed twice with PBS and then cultured in serum-free medium for 24 h before media collection to eliminate interference from serum proteases. Supernatants were cleared of debris by sequential centrifugation at 400×*g* for 5 min and 2000×*g* for 10 min at 4 °C. Tissue samples were homogenized in ice-cold assay buffer and cleared by centrifugation (10,000×*g*, 10 min, 4 °C). All cleared supernatants and tissue lysates were aliquoted and stored at − 80 °C until assay. Total protein concentration was determined by BCA assay. CTSD enzymatic activity was measured using a commercial CTSD activity kit (Cat. AB65302, Abcam, USA) according to the manufacturer’s protocol. CTSD activity was normalized to total protein content determined by BCA assay.

### Cell signaling pathway analysis in choroidal vascular cells

WT and *Tpc2*^−/−^ choroidal vascular cells were cultured in serum-free basal medium (EBM-2) for 24 h to synchronize the cells. Following serum starvation, cells were treated with 50 ng/mL VEGF (450-32-10UG, PeproTech, USA), with or without 200 ng/mL CHIL3(Cat. HY-P7845, MCE, USA), for various time points (0, 5, 15, 30, 60 min) to assess the VEGF and CHIL3-modulated signaling. After treatment, cells were lysed in RIPA lysis buffer with protease and phosphatase inhibitor cocktail. Protein concentrations were measured using a BCA assay. The Western blot was conducted as previously described. The membranes were incubated overnight at 4 °C with primary antibodies targeting phospho-NF-κB p65 (1:1000, Cat. 3303 T, CST, USA), NF-κB p65 (1:1000, Cat. 8242 T, CST, USA), phospho-P38 MAPK (1:1000, Cat. 9211, CST, USA), P38 MAPK (1:1000, Cat. 9212, CST, USA), phospho-ERK1/2 (1:1000, Cat. 9106, CST, USA), ERK1/2 (1:1000, Cat. 9102, CST, USA). After washing, membranes were incubated with HRP-conjugated secondary antibodies (1:2000, Cat.7074/7076, CST, USA) for 1 h at RT. Protein expression levels of phosphorylated and total NF-κB, P38 MAPK, and ERK1/2 were quantified using densitometry with Image Lab software (Bio-Rad Laboratories).

### **Validation of sgRNA targeting murine*Tpc2***

Plasmids encoding sgRNAs targeting the murine *Tpc2* coding region were designed using CHOPCHOP and synthesized. The sequences used were sgTpc2-1: ACCGATTGCCGCACTCAGGA and sgTpc2-3: TGGCCTGACCGAGACGATCG. Plasmids were introduced into HEK-msTPC2-GFP cells [18], and genome editing was validated by Sanger sequencing of PCR-amplified target loci. Indel formation efficiencies were quantified using Tracking of Indels by DEcomposition (TIDE) analysis, and cleavage at the expected sites was further confirmed by T7 endonuclease I (T7EI) assay and flow cytometry.

### Nucleofection of primary choroidal vascular cells

CRISPR-Cas9 plasmids were delivered into mouse primary choroidal vascular cells using the 4D-Nucleofector™ System (Lonza) with the Primary Cell 4D-Nucleofector™ X Kit (Cat. No. V4XP-2032) following the manufacturer’s instructions. Briefly, 1–2 × 10⁶ cells were resuspended in Nucleofector™ Solution and mixed with 2 µg endotoxin-free plasmid DNA (sgRNA: Cas9 = 1:1). The mixture was transferred to a nucleocuvette strip and subjected to electrical pulse (program CA-167). Cells were immediately cultured in pre-warmed medium on coated plates at 37 °C with 5% CO₂.

### AAV-mediated CRISPR–Cas9 gene targeting in retinal microglia

#### AAV production

A dual AAV system was used to deliver CRISPR–Cas9 components targeting *Tpc2*, as previously described [24]. A modified AAV1 capsid carrying the “GL” peptide insertion described in Pavlou et al. [25] was used. Briefly, HEK293T cells were cultured in DMEM supplemented with 10% FBS and transfected at ~ 70–80% confluency using a standard triple-plasmid transfection protocol using polyethyleneimine (PEI). At 72 h post-transfection, cells and culture supernatants were harvested and subjected to three freeze–thaw cycles to release viral particles. Crude lysates were clarified by centrifugation, and AAV particles were purified by iodixanol step-gradient ultracentrifugation. Viral fractions were collected, buffer-exchanged into PBS, and concentrated using centrifugal filter units. Viral genome titers were determined by quantitative PCR and stored at − 80 °C until use.

#### AAV transduction in retinal microglia

For in vitro transduction, primary retinal microglia were incubated with dual AAVs (sgRNA: Cas9 = 1:1) at the total viral genome of 2.5 × 10^10^ in microglia medium. Cells were maintained for an additional 7 days to allow for CRISPR-mediated gene editing.

### Generation of TPC2 KO iPSC and differentiation to endothelial cells

#### Culture and maintenance of iPSC

The induced pluripotent stem cell line B7-TetOn-ETV2.2, kindly provided by Prof. Dr. Volker Buskamp (Bonn, Germany) based on an iPSC clone described in C. S. Cowan et al. [26], was used in all experiments and is hereinafter referred to as iPSCs. Cells were cultured on Matrigel-coated 6-well plates in mTeSR™ Plus medium (STEMCELL Technologies, Canada). The medium was changed every two days by fully aspirating the old medium and adding 1 mL of fresh medium. Cells were maintained at 37 °C, 5% CO_2_. Once achieved 70–80% of confluency, cells were washed with Dulbecco’s balanced salt solution (D-PBS, Gibco, USA) and detached using ReLeSR™ (STEMCELL Technologies, Canada). For efficient detachment, 1 mL ReLeSR was added, incubated for 50 s, aspirated, and then incubated for another 4 min. After incubation, 0.5 mL fresh mTeSR™ Plus medium was added to the cells to detach them. This step was repeated with another 0.5 mL of mTeSR™ Plus medium. Cells were split at a 1:5 ratio and transferred to a Matrigel-coated 6-well plate containing 1 mL of fresh mTeSR™ Plus medium. After splitting, the medium was changed 24 h later.

#### ***TPC2*Knockout (KO) in iPSC**

Two guide RNAs (sgRNA-top and sgRNA-btm) targeting distinct sites within the human *TPC2* coding region were designed (CHOPCHOP) and synthesized (sequences: sgRNA-top: CCATTTCCGGCAGCGACCAG, sgRNA-btm: ACTGGACAGAGTCCGCACAT) (Integrated DNA Technologies, USA). Alt-RTM CRISPR-Cas9 Trans-Activating crRNA ATTOTM 550 (tracrRNA, Integrated DNA Technologies, USA) was complexed with the two sgRNAs to form ribonucleoprotein (RNP) complexes following the manufacturer’s instructions (sgRNA: Cas9 molar ratio typically 2:1). RNPs were introduced into iPSCs by nucleofection using Amaxa 4D Nucleofector (Lonza, Switzerland) with P3-nucleofection solution optimized for human iPSCs (program CB-150). Cells were recovered in pre-warmed mTeSR1 supplemented with 10 µM ROCK inhibitor for 24 h and then expanded.

Single-cell clones were obtained by FACS cell sorting and screened by PCR across the targeted locus. Candidate clones showing altered amplicon size were subjected to Sanger sequencing to confirm the intended fragment deletion. Protein-level validation was performed by Western blot using anti-TPC2 antibody, with β-actin as loading control. A validated clone (clone 1B3) lacking detectable TPC2 protein was selected for downstream differentiation.

#### iPSC differentiation into iEC

iPSCs (WT and *TPC2*^−/−^) were differentiated toward the endothelial lineage using a stepwise protocol from Luo et al. [27]. For Stage 1 (mesodermal progenitor differentiation), cells were cultured in 2 mL basal medium supplemented with 6 µM CHIR99021 (Sigma Aldrich, USA); the basal medium consisted of Gibco™ Advanced DMEM/F12 Medium (Thermo Fisher Scientific, USA), 1× GlutaMAX™ Supplement (Thermo Fisher Scientific, USA), 60 µg/mL L-Ascorbic acid phosphate, and 0.4× P/S. Medium was refreshed after 24 h, and mesodermal progenitor cells were obtained after another 24 h. For Stage 2 (endothelial specification), mesodermal progenitor cells were detached with 0.5 mL Trypsin-EDTA, neutralized, and seeded onto Matrigel-coated dishes in S2 medium, consisting of the same basal medium supplemented with 0.5 µg/mL doxycycline hyclate, 10 ng/mL hEGF (Cat. AF-100-15-100UG, PeproTech, USA), 50 ng/mL hFGF-2 (Cat. AF-100-18B-50UG, PeproTech, USA), and 50 ng/mL VEGF (Cat. 100-20-50UG, PeproTech, USA). Medium was refreshed after 24 h, and iECs were obtained after another 24 h. From day 5 onward, cells were maintained in EGM-2 medium. Endothelial identity was confirmed by immunostaining for vWF (1:200, Cat. sc-53466, SantaCruz Biotechnology, USA) and VE-cadherin (1:200, Cat. sc-9989, SantaCruz Biotechnology, USA). Functional loss of TPC2 in KO iECs was confirmed by lysosomal patch-clamp recording showing absence of TPC2-mediated currents followed the process described by Chen et al. [28].

### Statistical analysis

Data are presented as mean ± standard error of the mean (SEM). Student’s t-test was used to compare two different groups. Additionally, a one-way analysis of variance (ANOVA) was conducted, followed by post hoc Dunnett’s tests for multiple comparisons. Two-way ANOVA was used for analyses involving two independent variables, followed by Tukey’s multiple comparisons test. A *p*-value < 0.05 was considered statistically significant. The graphs were generated by Prism 10.0 and figures with Affinity designer 2.

## Results

### *Tpc2* deletion downregulates pro-angiogenic factors in retinal microglia

**Fig. 1:**
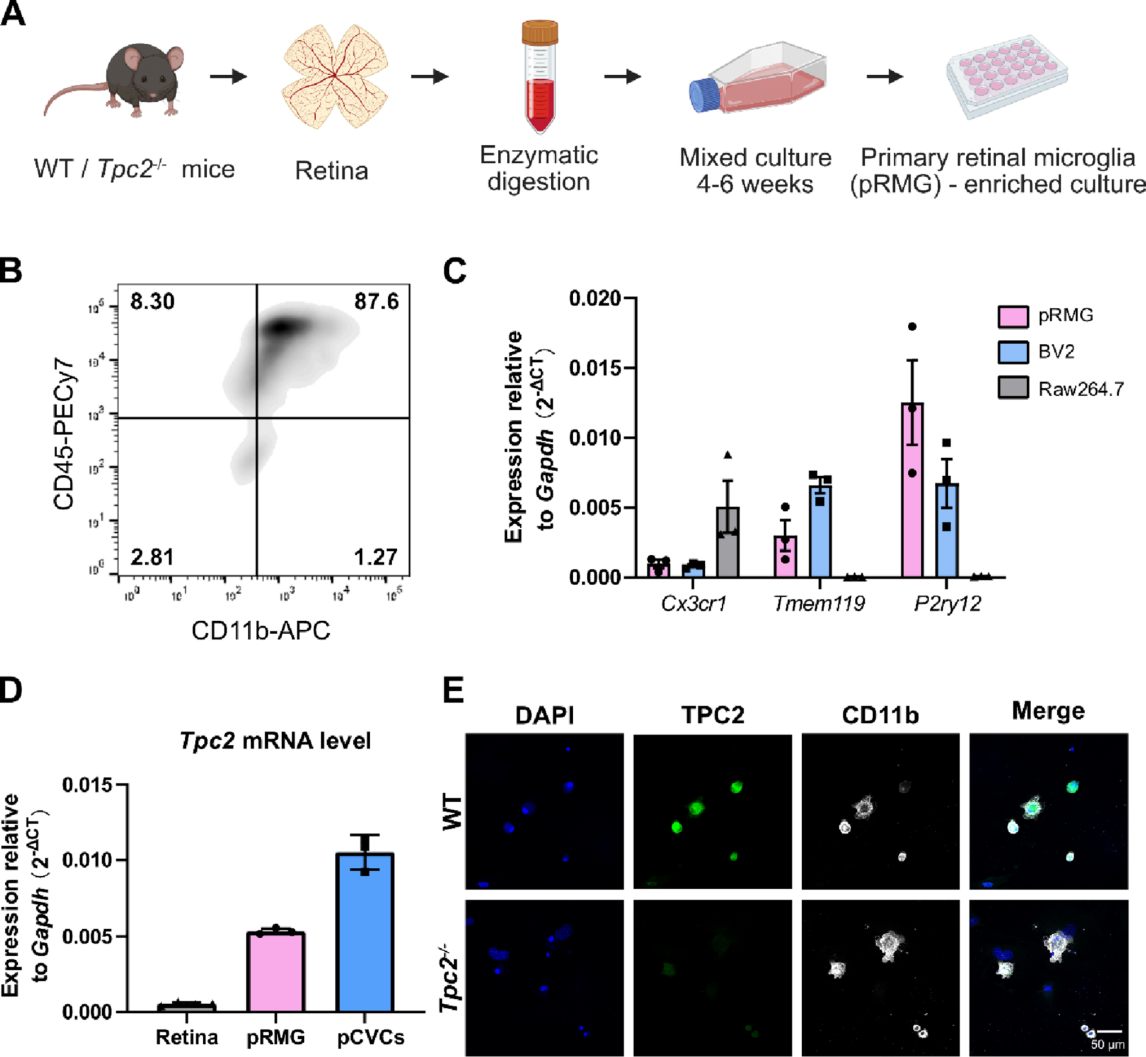
Characterization of primary retinal microglia-enriched culture. **A** Schematic workflow illustrating the isolation of primary retinal microglia- enriched (pRMG) culture. **B** Flow cytometry characterization of pRMG showing high level of CD45 and CD11b expression. **C** qRT-PCR analysis of myeloid (*Cx3cr1*) and microglial markers (*Tmem119*,* P2ry12*) in pRMG, BV2 cells, and RAW264.7 cells. **D** Relative *Tpc2* mRNA expression in mouse retina, pRMG and primary choroidal vascular cells (pCVCs). Data are presented as mean ± SEM. **E** Representative immunofluorescence images showing TPC2 expression in CD11b⁺ pRMG from WT and *Tpc2*^⁻/⁻^ mice (TPC2, green; CD11b, white; and DAPI, blue). Illustration in panel (**A**) was generated with biorender.com

*Tpc2* is required for endolysosomal cytokine release in immune cells (retinal microglia and macrophage), and its loss leads to impaired IL-1β secretion and reduced neovascularization in the CNV mouse model of nAMD [17]. However, it has remained unclear whether TPC2 regulates additional microglia-derived factors beyond IL-1β, particularly those involved in maintaining retinal immune homeostasis and modulating angiogenic signaling. To investigate the molecular consequences of *Tpc2*-deficiency in retinal microglia, we isolated and expanded primary retinal microglia (pRMG) in vitro (Fig. 1A). The purity of the retinal microglia-enriched cultures was confirmed by flow cytometry, with more than 85% of cells co-expressing CD45 and CD11b, consistent with a microglial identity (Fig. 1B). qRT-PCR analysis further showed *Tmem119* and *P2ry12* were highly expressed in both pRMG and BV2 microglia cells, whereas their expression was nearly undetectable in RAW 264.7 macrophage cells, consistent with a microglia-associated gene expression signature distinct from macrophage cells (Fig. 1C). Meanwhile, *Cx3cr1* was expressed across all three groups, consistent with its known broad expression within the myeloid system [29]. Moreover, *Tpc2* mRNA expression is substantially more enriched in pRMG compared to whole retinal tissue (Fig. 1D). Immunofluorescence staining validated TPC2 protein expression in pRMG (Fig. 1E).

**Fig. 2:**
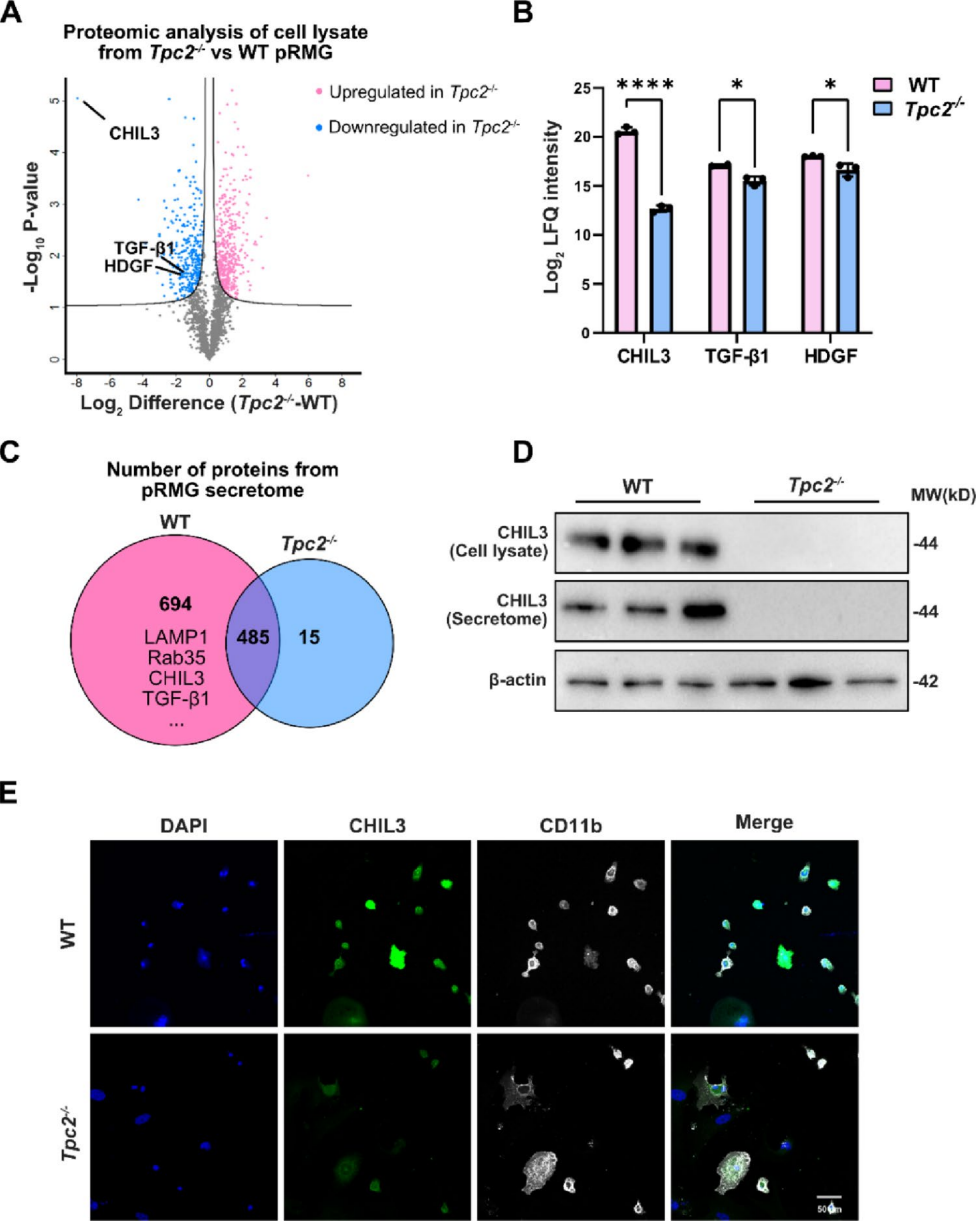
Decreased pro-angiogenic factors in *Tpc2*^⁻/⁻^ retinal microglia and their secretomes. **A** Volcano plot showing differentially expressed cellular proteins between WT and *Tpc2*^⁻/⁻^ primary retinal microglia (pRMG) (*n* = 3). **B** Absolute protein abundance of representative pro-angiogenic factors (CHIL3, TGF-β1, and HDGF) from WT and *Tpc2*^⁻/⁻^ pRMG cell lysates. Two-tailed t test was used for statistical analysis. **C** Venn diagram showing the number of detectable proteins secreted into the culture supernatant (secretome) of WT and *Tpc2*^⁻/⁻^ retinal microglia. **D** Western blot analysis of CHIL3 expression in cell lysates and culture supernatant from WT and *Tpc2*^⁻/⁻^ retinal microglia cultures. β-actin was used as a loading control for the cell lysates. **E** Representative immunofluorescence images showing CHIL3 expression in CD11b⁺ retinal microglia from WT and *Tpc2*^⁻/⁻^ mice. Data are presented as mean ± SEM. (*p < 0.05, ****p < 0.0001).

Next, we sought to determine how *Tpc2*-deficiency influences the molecular landscape of pRMG. Proteomic profiling of *Tpc2*^⁻/⁻^ retinal microglia revealed numerous differentially expressed proteins compared to WT (Fig. 2A). Several pro-angiogenic factors were downregulated in *Tpc2*^⁻/⁻^ pRMG, including chitinase-like protein 3 (CHIL3), transforming growth factor beta 1 (TGF-β1), and hepatoma-derived growth factor (HDGF) (Fig. 2B) as representative. Given that TPC2 has been implicated in endolysosomal trafficking and vesicle fusion, processes that are essential for regulated cytokine secretion, we next examined whether the loss of TPC2 also alters the secretome of retinal microglia. Mass spectrometry identified 485 proteins common to both WT and *Tpc2*^⁻/⁻^ secretomes. Strikingly, 694 unique proteins were only detected in WT microglia supernatants, whereas only 15 were uniquely detectable in the *Tpc2*^⁻/⁻^ group. Fisher’s exact test indicated the difference in the number of uniquely detected proteins (odds ratio = 32.3, *p* < 1 × 10⁻⁹⁵), indicating altered protein detection profiles in *Tpc2*-deficient microglia secretome under the applied analytical criteria. While stochastic sampling and threshold-dependent detection effects may contribute to apparent differences for low-abundance proteins, the substantial overlap of the core secretome supports the robustness of the observed genotype-dependent changes. Notably, proteins associated with vesicular and lysosomal compartments, for example LAMP1 and Rab35, can be only detected in supernatants of WT pRMG. It may indicate an altered vesicle-associated protein release profiles in the absence of *Tpc2* (Fig. 2C). Among the proteins of interest, pro-angiogenic factor CHIL3 showed the most pronounced difference in both intracellular and extracellular fractions of pRMG, therefore it was chosen for subsequent validation and in-depth characterization. Western blot analysis demonstrated undetectable CHIL3 signal in both lysates and secretome from *Tpc2*^⁻/⁻^ pRMG cultures (Fig. 2D). Immunofluorescence further confirmed markedly diminished CHIL3 signal in CD11b⁺ retinal microglia of *Tpc2*^⁻/⁻^ mice (Fig. 2E).

### Loss of TPC2 affects angiogenic signaling response in choroidal vascular cells

**Fig. 3:**
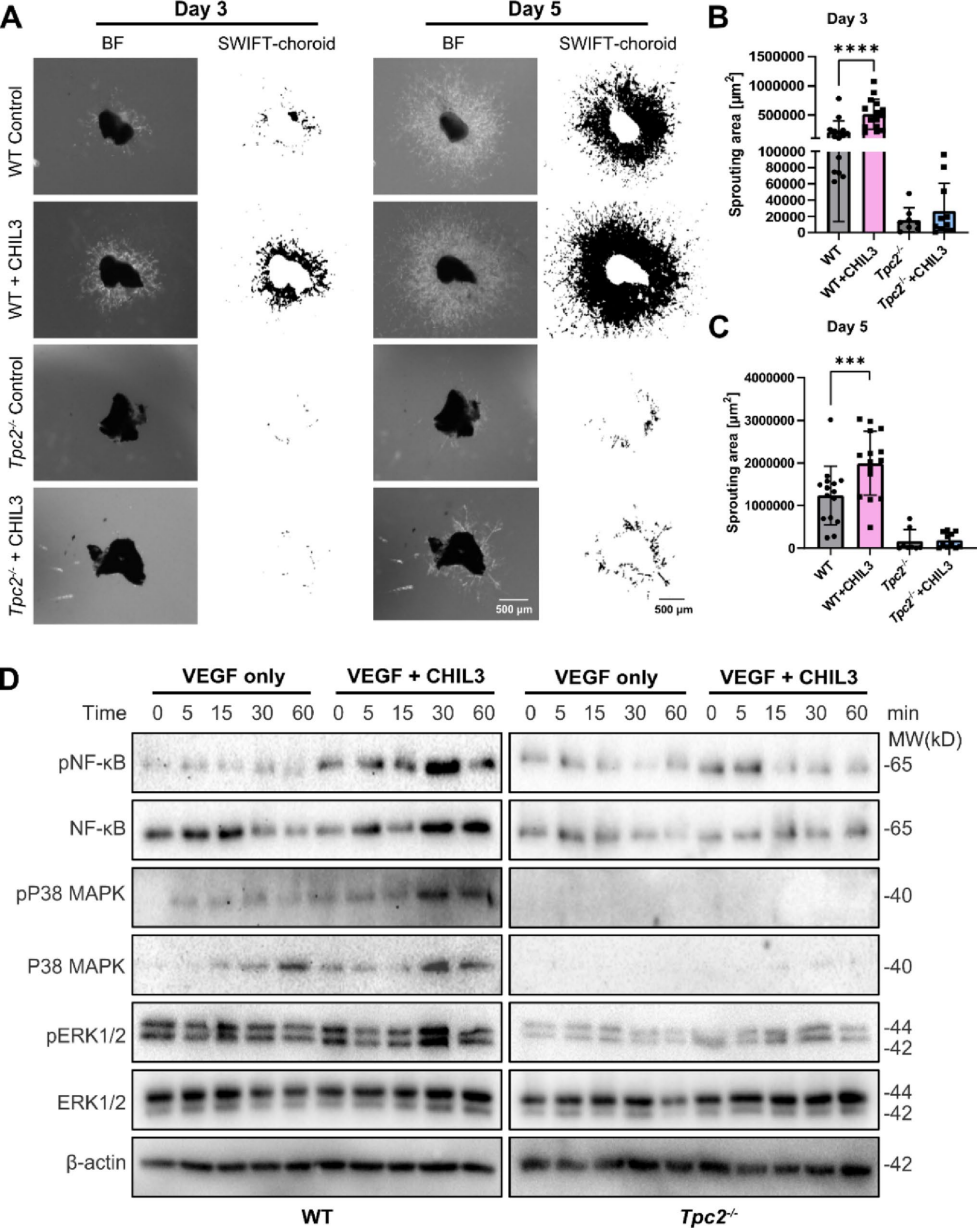
*Tpc2*-deficiency modulated choroidal sprouting and NF-κB/MAPK signaling responses. **A** Representative images of the choroidal sprouting assay on day 3 and day 5 under four conditions: WT, WT + CHIL3 (200 ng/mL), *Tpc2*^*−/−*^, and *Tpc2*^−/−^ + CHIL3 (200 ng/mL) (*n* ≥ 8). **B, C** Quantification of the sprouting area on day 3 (B) and day 5 (**C**). **D** Western blot analysis of NF-κB, P38 MAPK, ERK1/2 and their phosphorylated forms in choroidal vascular cells from WT and *Tpc2*^⁻/⁻^ mice. Cells were treated with VEGF (50 ng/mL) or VEGF (50 ng/mL) combined with CHIL3 (200 ng/mL) for 0 to 60 min (min). β-Actin was used as the loading control. Experiments were independently repeated three times. Two-way ANOVA was used for statistical analysis. Data are presented as mean ± SEM. (***p < 0.001; ****p < 0.0001)

To investigate how the loss of TPC2 influences angiogenic responses in choroidal vascular cells, choroidal sprouting assays from WT and *Tpc2*^⁻/⁻^ choroidal explants were performed. As previously shown, neovascularization in this choroidal explant model depends on TPC2 [17]. Because of its pronounced differential downregulation in *Tpc2*^⁻/⁻^ retinal microglia, we selected CHIL3 among the identified pro-angiogenic factors for subsequent functional assays. To this end, we examined whether the addition of CHIL3 affects neovascularization induced by EBM-2 complete medium in choroidal explants (see Methods for details). Supplementation of WT choroidal explants with 200 ng/mL recombinant CHIL3 protein enhanced sprouting at both day 3 (fold change: 2.51, *p* < 0.0001) and day 5 (fold change: 1.61, *p* = 0.0009) compared with explants that received the standard treatment (Fig. 3A). In contrast, *Tpc2*^⁻/⁻^ explants exhibited markedly reduced sprouting with the standard treatment (fold change at day 3: 0.08, *p* = 0.022; fold change at day 5: 0.13, *p* = 0.0001), and co-application of CHIL3 failed to rescue this TPC2-related impairment in neovascularization (fold change at day 3: 1.71, *p* = 0.90; fold change at day 5: 1.37, *p* = 0.93) (Fig. 3B, C). These results indicate that exogenous CHIL3 can enhance angiogenic responses in WT choroidal explants but is insufficient to compensate for the loss of TPC2 in *Tpc2*^⁻/⁻^ explants.

To explore the signaling mechanisms underlying the TPC2-dependent neovascularization, we examined key angiogenic-related pathways in primary choroidal vascular cells isolated from sprouting choroid explants by brief trypsinization, filtration, centrifugation, and resuspension (Fig. S1B). The endothelial identity of pCVCs was verified by flow cytometry-based confirmation of co-expression of Endomucin and CD31 (Fig. S1C). pCVCs were stimulated with VEGF (50 ng/mL) alone or in combination with CHIL3 (200 ng/mL). Western blot analysis was performed to assess the activation of NF-κB and MAPK signaling pathways (ERK1/2 and P38 MAPK) (Fig. 3D). Statistical analysis was performed by Two-Way ANOVA. In WT pCVCs, NF-κB signaling can be significantly enhanced by CHIL3 co-stimulation compared with only VEGF treatment at 30 min (*p* = 0.046). ERK1/2 phosphorylation levels were in gerneral lower in *Tpc2*^*⁻/⁻*^ cells than in WT cells both in VEGF only and VEGF+CHIL3 treatment conditions. Total P38 MAPK level was reduced in *Tpc2*^⁻/⁻^ cells in VEGF only and VEGF+CHIL3 groups, while phosphorylated P38 MAPK was barely detectable. Furthermore, neither VEGF alone nor VEGF+CHIL3 treatment elicited a significant activation response of the NF-κB, ERK1/2, or P38 MAPK signaling pathways in *Tpc2*^⁻/⁻^ cells (Fig. S2). This indicated an association between attenuated angiogenic signaling responsiveness and the absence of TPC2.

Having established that microglia-derived pro-angiogenic factors synergize with VEGF to drive pathological angiogenesis, we next asked whether the microglial secretome acts through TPC2-dependent mechanisms to further regulate pCVCs behavior. pRMG were cultured until confluence and then maintained in serum-free basal medium for 48 h to generate retinal microglia conditioned medium (CM). After removing debris by centrifugation, the pRMG CM was applied to pCVCs for an additional 48 h to investigate effects at the level of gene expression and proteome (Fig. S3A). qRT-PCR analysis showed that pCVCs treated with CM from WT pRMGs exhibited a significant upregulation of *Il6* gene expression compared to untreated control pCVCs, indicating a strong inflammatory response induced by the secretome of WT pRMGs. However, when *Tpc2*^⁻/⁻^ pCVCs were treated with CM from *Tpc2*^⁻/⁻^ pRMG, *Il6* remained unchanged, suggesting that TPC2 is required for the induction of microglia-driven inflammatory signaling in pCVCs. Other genes including *Vegfa*, *Vegfr2*, *Ccl2*, and *Icam1* showed a similar but less pronounced TPC2-dependent decrease in expression (Fig. S3B).

Proteomic profiling further revealed distinct protein expression patterns in choroidal vascular cells. WT pCVCs treated with CM from WT vs. *Tpc2*^⁻/⁻^ pRMG displayed downregulation of proteins mainly involved in lysosomal function and extracellular matrix pathways, as illustrated by heatmap clustering (Fig. S3C). CM from WT pRMG selectively induced the levels of a number of homeostasis proteins associated with metabolic, lysosomal, antioxidant, and stress-related pathways in WT pCVCs, effects that were weaker when CM from *Tpc2*^⁻/⁻^ pRMG was used instead (Fig. S3C). Representative examples include lysosomal enzymes (e.g., HEXB, α-NAGA, GLB1), lipid metabolism-related proteins (e.g., ABCA1, LIPA), antioxidant/homeostasis regulators (e.g., VNN1, ZFAND6), and stress/apoptosis-related proteins (e.g., MLKL, FAS). These data suggest that *Tpc2*-deficiency in microglia is associated with altered secretion of factors that influence endothelial homeostasis and lysosomal responses. Notably, WT pRMG CM robustly activated a lysosomal program in pCVCs, evidenced by increased expression of representative hydrolases (e.g., CTSB, GUSB), lysosomal membrane proteins (e.g., LAMP1, SCARB2), and mannose-6-phosphate trafficking receptors (e.g., IGF2R) (Fig. S3C). The upregulation of these lysosomal-related proteins was absent in *Tpc2*^⁻/⁻^ pCVCs, suggesting that TPC2 contributes to the responsiveness of choroidal vascular cells to microglia-derived signals regulating lysosomal pathways. Furthermore, WT pRMG CM selectively promoted a cohort of proteins associated with cytoskeletal dynamics, focal adhesion, integrin signaling, and pro-angiogenic kinase cascades, effects that were not observed in *Tpc2*^⁻/⁻^ pCVCs. Representative proteins include small GTPases and actin regulators (e.g., RHOA, RAC1), focal adhesion/adaptor proteins (e.g., VCL, TLN2), integrins and ECM receptors (e.g., ITGA3, ITGB1), and signaling kinases (e.g., EGFR, MAPK1/3, AKT2) (Fig. S3C). These findings indicate that WT pRMG CM is associated with the induction of proteins involved in cell migration, adhesion, and angiogenesis, including components of integrin-focal adhesion and MAPK/PI3K-AKT signaling pathways in pCVCs. The attenuated response observed in *Tpc2⁻/⁻* pCVCs suggests that TPC2 contributes to the activation of these angiogenesis-related signaling programs.

### TPC2 regulates lysosomal cathepsin-mediated choroidal neovascularization

To determine whether TPC2 regulates neovascularization through cell-intrinsic mechanisms in choroidal vascular cells, we compared *Tpc2* expression in pCVCs and pRMG by qRT-PCR. The analysis revealed that pCVCs express higher levels of *Tpc2* than pRMG (Fig. 1D). Consistently, *Tpc2* mRNA was more abundant in the RPE/choroid than in the retina of WT mice at 1, 3, and 10 months of age, with no significant age-dependent variation (Fig. S1C). These findings indicate that *Tpc2* is constitutively expressed and preferentially enriched in the choroidal compartment of the eye. Under physiological conditions, the function of TPC2 can be regulated in both directions via cell intrinsic signaling. For example, TPC2 is activated by phosphatidylinositol 3,5-bisphosphate (PI(3,5)P2) or by nicotinic acid adenine dinucleotide phosphate (NAADP) and inhibited by ATP/mTOR signaling [30]. We therefore asked how pharmacological inhibition and activation of TPC2 affects neovascularization in the choroidal sprouting assay. To inhibit TPC2, we used SG-094, a previously reported small-molecule TPC2 inhibitor [31]. Analysis revealed that SG094-treated samples showed sprouting areas which were about 43% (*p* = 0.34) and 12% (*p* = 0.0012) of the corresponding WT choroidal explants on day 4 and day 6, respectively (Fig. S4). To evaluate the impact of TPC2 activation on neovascularization, we treated WT choroidal explants with the established TPC2 agonists A1P [32]. Compared to control, the A1P treatment group showed an increased sprouting area on day 4 and day 6 (Fig. S4). On day 4, A1P induced an approximately 2.43-fold increase (*p* = 0.011). Similarly, on D6, A1P enhanced sprouting by about 1.81-fold compared to control (*p* = 0.0037). These results confirm that changes in TPC2 activity can have a direct impact on the angiogenic capacity of choroidal blood vessels.

**Fig. 4:**
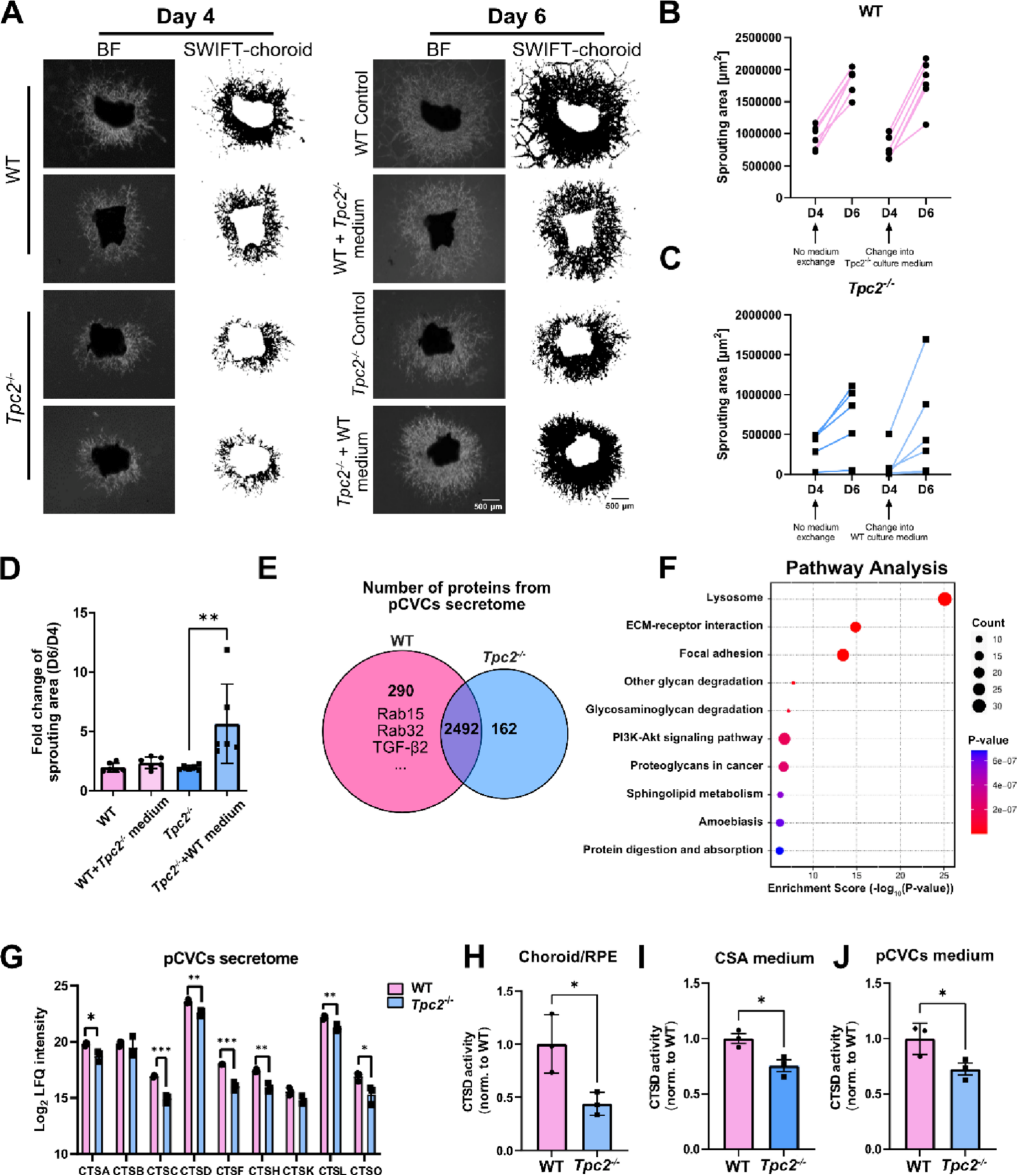
*Tpc2*-deficiency impairs choroidal sprouting by reducing lysosomal cathepsins secretion and activity. **A** Choroid explants from WT and *Tpc2*^−/−^ mice were cultured in Matrigel for 4 days. After 4 days, the culture media were exchanged between the WT and *Tpc2*^−/−^ explants, followed by an additional 2 days of culture before imaging and analysis. Representative images of choroidal sprouting from WT and *Tpc2*^−/−^ explants on day 4 and day 6 are shown. **B, C** Sprouting area of WT and *Tpc2*^−/−^ explants with or without media exchange, shown as paired data between day 4 and day 6. **D** Fold change of sprouting area between day 6 and day 4 for each group. Two-way ANOVA was used for statistical analysis. **E** Venn diagram showing the number of proteins detected in the secretome of WT and *Tpc2*^⁻/⁻^ primary choroidal vascular cells (pCVCs) (*n* = 3). **F** Pathway enrichment analysis of proteins downregulated in *Tpc2*^⁻/⁻^ secretome revealed significant alterations in lysosome-associated pathways. **G** Mass spectrometry-based label-free quantification (LFQ) of cathepsin family proteins in the conditioned medium of WT and *Tpc2*^⁻/⁻^ pCVCs. Two-tailed t test was used for statistical analysis. **H–J** CTSD activity in choroid/RPE lysates, culture medium of choroidal sprouting assay (CSA) and culture medium of pCVCs. Two-tailed t test was used for statistical analysis. Data are presented as mean ± SEM. (*p < 0.05; **p < 0.01; ***p < 0.001).

Next, we examined whether TPC2 affects neovascularization through autocrine signaling of the choroidal tissue. To determine whether soluble factors released from the choroid contribute to the sprouting phenotype, we performed a media exchange assay. Choroid explants from WT and *Tpc2*^⁻/⁻^ mice were cultured in Matrigel for 4 days, after which the culture media were swapped between groups and cultures were maintained for 2 additional days (Fig. 4A). Quantitative analysis of fold-change in sprouting area from day 4 to day 6 confirmed these observations: *Tpc2*^⁻/⁻^ explants exhibited a significantly greater increase when cultured with WT-culture medium (5.65 ± 3.33-fold) compared to their own medium (1.97 ± 0.19-fold, *p* = 0.0012), while WT explants showed comparable sprouting regardless of media condition (Fig. 4B, C,D). This experiment showed that WT- culture medium markedly enhanced sprouting in *Tpc2*^⁻/⁻^ explants, whereas *Tpc2*^⁻/⁻^- culture medium failed to suppress sprouting in WT explants. These findings suggest that WT choroid releases pro-angiogenic soluble factors that can partially rescue the defective sprouting in *Tpc2*^⁻/⁻^ choroidal tissue, implicating TPC2 in the regulation of secretory signaling.

To further investigate this possibility, we used mass spectrometry to analyze the secretome and proteome of pCVCs derived from WT and *Tpc2*^⁻/⁻^ mice. In total, we detected 2,492 proteins that were present in conditioned medium of both groups, while 290 and 162 proteins were uniquely identified in WT and *Tpc2*^⁻/⁻^ samples, respectively (Fig. 4E). Among the proteins detected only in WT cells were Rab15, Rab32 and TGF-β2, consistent with a TPC2-dependent regulation of vesicle-mediated secretion. Fisher’s exact test indicated that WT tissue contained a significantly higher proportion of unique proteins compared with *Tpc2*^⁻/⁻^ tissue (odds ratio = 1.79, *p* = 7.6 × 10⁻⁹). Pathway enrichment analysis confirmed significant alterations in lysosome-associated pathways (Fig. 4F). Among the most consistently reduced proteins were lysosomal hydrolases, particularly members of the cathepsin family (CTSA, CTSC, CTSD, CTSF, CTSH, CTSL, CTSO). The secretion of these enzymes was significantly diminished in *Tpc2*^⁻/⁻^ cells, suggesting defective lysosomal maturation and/or exocytosis in the absence of TPC2 (Fig. 4G). To functionally validate this finding, we quantified CTSD activity in choroid/RPE tissue lysates, as well as in culture medium from choroidal sprouting assays and pCVCs cultures. CTSD enzymatic activity was found to be reduced by 56% in choroid/RPE tissue lysates (*p* = 0.03), by 25% in choroidal sprouting assay supernatants (*p* = 0.02), and by 28% in pCVCs culture medium (*p* = 0.046) due to *Tpc2*-deficiency (Fig. 4H–J). These data collectively demonstrate that TPC2 contributes to lysosome-dependent secretion in vascular endothelial cells, and its loss leads to reduced extracellular release of proteolytic enzymes such as CTSD, thereby impairing the regulation of choroidal neovascularization.

### *Tpc2* knockout impairs the angiogenic capacity of choroidal vascular cells

Next, we aimed to investigate the effect of acute knockout of *Tpc2*. To this end, we first designed *Tpc2*-specific single guide RNAs (sgRNAs) using CHOPCHOP and CRISPOR and selected two candidates (sgTpc2-1, targeting Exon 17, and sgTpc2-3, targeting Exon 4) based on scoring results. We then validated their CRISPR–Cas9-mediated targeting efficiency in msTPC2-GFP reporter HEK cells. Sanger sequencing chromatograms revealed mixed peaks downstream of the CRISPR-Cas9 cut sites, consistent with frameshift mutations (Fig. S5A). Indel formation efficiencies quantified by Tracking of TIDE were 64.5% for sgTpc2-1 and 40.5% for sgTpc2-3 (Fig. S5B). T7EI assays further confirmed cleavage at the expected loci (Fig. S5C), and flow cytometry revealed a marked reduction in TPC2-GFP signal, validating effective loss of TPC2 protein expression (Fig. S5D).

**Fig. 5:**
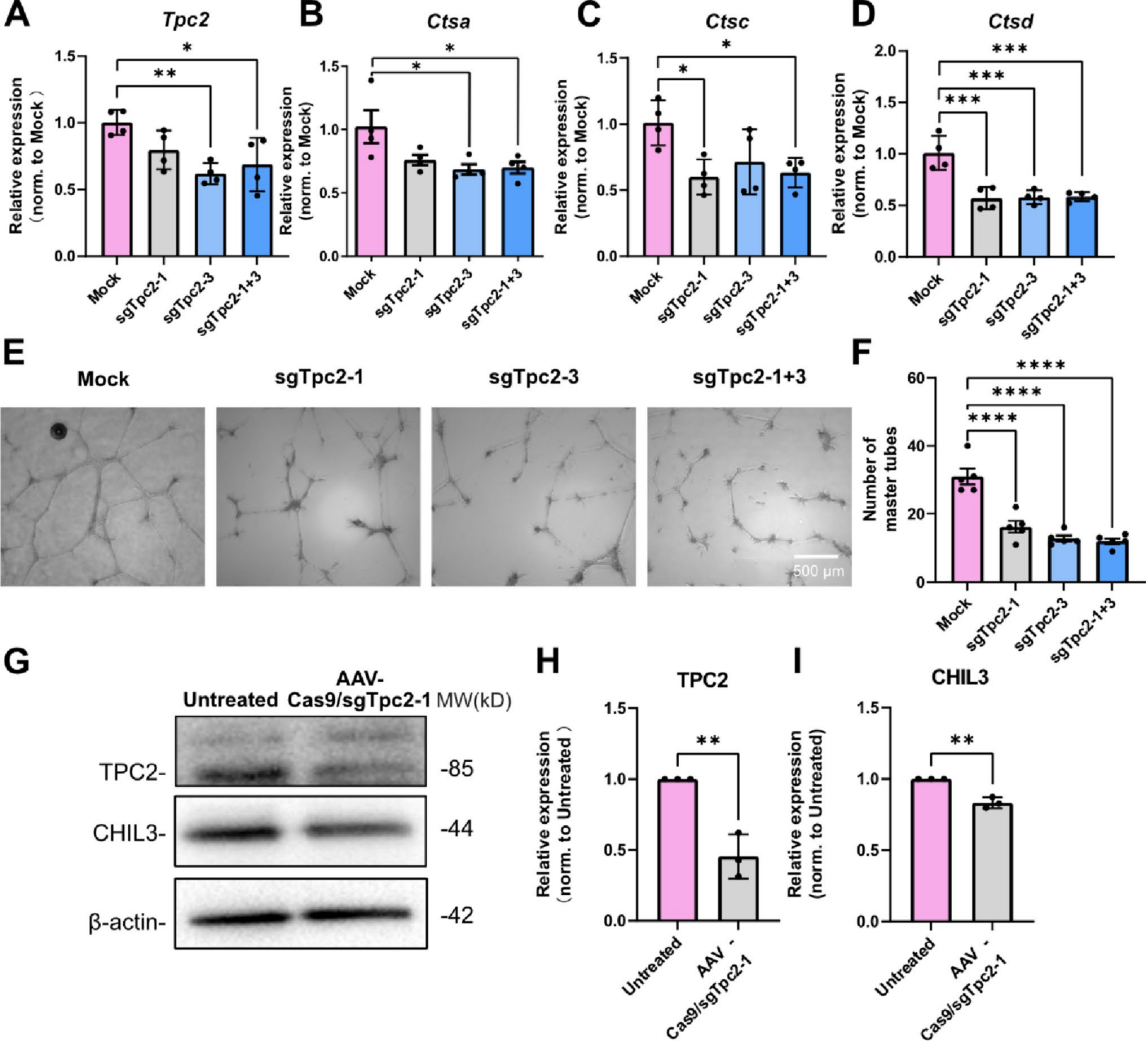
Effects of CRISPR/Cas9-mediated *Tpc2* knockout in choroidal vascular cells and retinal microglia. **A** *Tpc2* mRNA levels in primary choroidal vascular cells following CRISPR/Cas9-mediated targeting of *Tpc2* (*n* = 4). **B–D** Relative mRNA expression levels of lysosomal genes *Ctsa*, *Ctsc*, and *Ctsd* after CRISPR/Cas9-mediated *Tpc2* targeting (*n* = 4). **E** Representative images of tube formation assays acquired using an EVOS microscope 16 h after CRISPR/Cas9-mediated nucleofection (*n* = 5). **F.** Quantification of master tube numbers from tube formation assays. **G** Representative western blot analysis of retinal microglia after transduction. The blots were probed for TPC2 and CHIL3, with β-actin used as a loading control (*n* = 3). **H** Quantification of *Tpc2* knockout efficiency based on Western blot analysis. **I** Densitometric analysis of CHIL3 protein expression following TPC2 knockout. One-way ANOVA and Two-tailed t test were used for statistical analysis. Data are presented as mean ± SEM. (*p < 0.05, **p < 0.01, ***p < 0.001)

Building on this validation, we next assessed the functional consequences of TPC2 loss in pCVCs. CRISPR–Cas9-mediated knockdown was achieved via nucleofection, and qRT-PCR analysis revealed significant reduction in *Tpc2* mRNA following delivery of sgTpc2-3 alone or in combination with sgTpc2-1 (Fig. 5A). To explore whether *Tpc2* depletion is associated with changes in lysosome-related pathways, we examined the expression of lysosomal regulators associated with TPC2, including *Ctsa*, *Ctsc*, and *Ctsd*, all of which were reduced to varying degrees upon partial *Tpc2* knockout (Fig. 5B-D). Functional assessment by tube formation assay revealed impaired capillary-like network formation in *Tpc2*-targeted groups compared to mock controls (Fig. 5E), with quantitative analysis confirming a marked reduction in the number of master tubes across all CRISPR-treated conditions (Fig. 5F). These findings indicate that TPC2 contributes to the angiogenic capacity of pCVCs and that partial loss of TPC2 is associated with impaired capillary-like network formation.

We next examined the effects of acute *Tpc2* knockout in retinal microglia. Because pRMG are refractory to efficient nucleofection, we employed an AAV-based CRISPR–Cas9 delivery strategy to achieve gene knockout. We used sgTpc2-1 shown to have the highest editing efficiency in HEK cells. Western blot analysis confirmed a robust reduction of TPC2 protein levels in pRMG following AAV vector administration (Fig. 5G), with densitometric quantification indicating an average knockdown efficiency of 54.5% (Fig. 5H). Notably, TPC2 knockout in pRMG was accompanied by a concomitant decrease in CHIL3 protein expression (16.8%), as demonstrated by Western blotting and densitometric analysis (Fig. 5I). These findings demonstrate that partial reduction of TPC2 in pRMG is feasible and is associated with reduced CHIL3 expression, further supporting a potential role for TPC2 in regulating microglia-associated pro-angiogenic signaling.

### *TPC2* knockout in iPSC-derived endothelial cells leads to impaired angiogenic capacity

**Fig. 6:**
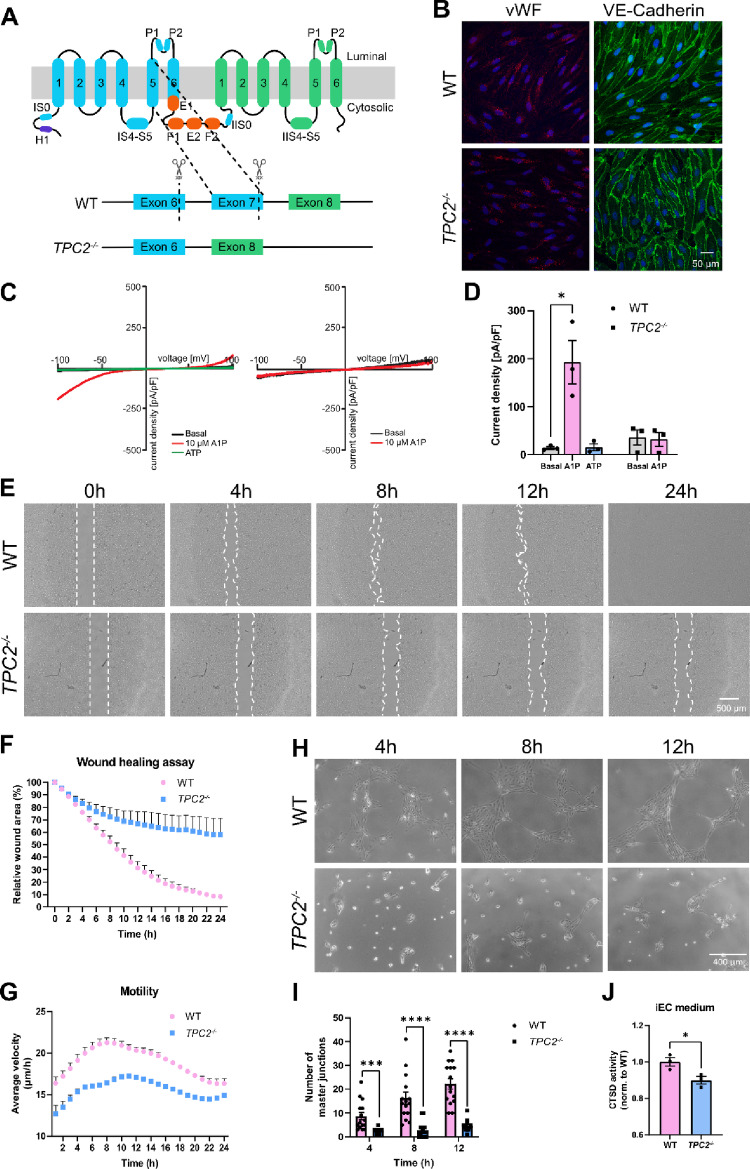
*TPC2*-deficiency impairs the angiogenic activity of iPSC-derived endothelial cells. **A** Schematic representation of the CRISPR/Cas9 targeting strategy used for the knockout of *TPC2* in human iPSCs. **B** Immunofluorescence staining of iPSC-derived endothelial cells (iECs) showing expression of endothelial cell markers vWF (red) and VE-cadherin (green). **C** Lysosomal patch-clamp recordings of TPC2-mediated currents (induced by A1P) in WT and *TPC2*^⁻/⁻^ iECs. **D** Quantification of lysosomal currents showing functional loss of TPC2 activity in knockout iECs. **E** Representative images of wound healing assay from 0 to 24 h (scratch width: 500 μm) (*n* = 3). **F** Quantification of wound closure in WT and *TPC2*^⁻/⁻^ iECs. **G** Quantification of cell motility in WT and *TPC2*^⁻/⁻^ iECs during culture. **H** Representative images of tube formation assay from WT and *TPC2*^⁻/⁻^ iECs at 4, 8, and 12 h. **I** Quantification of tube formation from WT and *TPC2*^⁻/⁻^ iECs. **J** Measurement of CTSD activity in culture medium of iECs. Two-tailed t test was used for statistical analysis. Data are presented as mean ± SEM. (*p < 0.05, ***p < 0.001, ****p < 0.0001)

In order to extend our findings from mice to humans, we engineered human iPSCs genetically using the CRISPR/Cas9 system to disrupt the *TPC2* gene. PCR genotyping and Sanger sequencing confirmed the desired deletion within the *TPC2* coding region (Fig. 6A and Fig. S6A-B), while Western blot analysis verified the complete loss of TPC2 protein expression in multiple clones (Fig. S6C). A validated clone (clone 1B3) lacking detectable TPC2 protein was selected for downstream differentiation. Following differentiation according to a stepwise endothelial induction protocol [27] (Fig. S6D), both WT and *TPC2*^*−/−*^ iPSCs successfully generated iECs with typical cobblestone morphology and positive staining for endothelial markers vWF and VE-cadherin (Fig. 6B). Compared with WT iECs, in which A1P activates TPC2 currents and (adenosine triphosphate) ATP inhibits this activation, lysosomal patch-clamp recordings revealed the absence of TPC2-mediated currents in *TPC2*^*−/−*^ iECs, confirming functional inactivation of the channel. (Fig. 6C, D).

In wound-healing assays, *TPC2*^*−/−*^ iECs displayed markedly impaired migration. After 24 h, the residual scratch area in KO cells was 58.0 ± 13.0% compared with 8.33 ± 1.20% in WT cells (*p* = 0.03) (Fig. 6E, F), reflecting impaired migratory capacity. Analysis of iEC motility during culture showed a generally lower average velocity of *TPC2*-deficient cells (Fig. 6G). Tube formation assays revealed a pronounced deficit in vascular-like network formation in *TPC2*^*−/−*^ iECs (Fig. 6H). Quantification of the number of master junctions demonstrated a progressive divergence between groups over time: at 4 h, WT 8.67 ± 0.89 vs. KO 2.60 ± 0.51 (*p* < 0.001); at 8 h, WT 16.27 ± 1.44 vs. KO 2.80 ± 0.62 (*p* < 0.0001); and at 12 h, WT 22.27 ± 2.03 vs. KO 4.64 ± 0.81 (*p* < 0.0001; Fig. 6I). This corresponds to a 70–80% reduction across time points (4–12 h) in *TPC2*-deficient cells relative to WT. Finally, to validate the effect of TPC2 on CTSD across species, we examined the enzymatic activity of CTSD in *TPC2*^*−/−*^ iEC culture medium and found a significant decrease of approximately 10% compared to WT (*p* = 0.046) (Fig. 6J), partially recapitulating the lysosomal alterations observed in the mouse model. Collectively, these data demonstrate that TPC2 contributes to angiogenic behavior in human iPSC-derived endothelial cells.

## Discussion

nAMD represents a multifactorial disease in which pathological choroidal angiogenesis is driven by intertwined vascular, inflammatory, and metabolic processes [33]. Anti-VEGF therapies remain the standard of care and provide a clinical benefit to many patients. However, the demand for repeated administration and variable efficacy in some patients due to therapy resistance highlights the need to identify novel molecular regulators that operate beyond canonical VEGF signaling [34]. In this context, lysosomal Ca²⁺ signaling has emerged as an important modulator of angiogenesis and immune activation. TPC2, a lysosomal two-pore Ca²⁺ channel, has previously been implicated in endolysosomal trafficking and endothelial function [35].Moreover, TPC2 has been shown to participate directly in VEGF/VEGFR2-mediated angiogenic signaling, via NAADP-triggered Ca²⁺ release from endolysosomal stores [36]. However, its precise contribution to retinal microglia-mediated inflammation and choroidal neovascularization remained unclear.

Our study identifies TPC2 as an important regulator of choroidal neovascularization and uncovers its roles in microglial function and choroidal angiogenesis. Genetic deletion or pharmacological inhibition of TPC2 in mice resulted in a marked reduction of angiogenic responses, whereas TPC2 activation enhanced vascular sprouting in choroidal explants. These observations highlight TPC2 as a modulator of the neovascular microenvironment and suggest its future potential as a therapeutic target in nAMD. To delineate the underlying mechanisms, we investigated two major cell types (retinal microglia and choroidal vascular cells), which represent the immune and endothelial arms of neovascular regulation respectively. By combining quantitative proteomics and functional confirmations, we aimed to dissect how TPC2 loss impacts microglial secretory function and endothelial angiogenic competence, thereby linking lysosomal functions and pathological angiogenesis.

Retinal microglia, the resident immune sentinels of the retina, are essential for immune surveillance, debris clearance, and maintenance of the neurovascular unit [37]. However, the roles of TPC2 in retinal microglia remained unexplored. We established an optimized and reproducible long-term culture system that overcomes the limited yield of primary retinal microglia, enabling the generation of sufficient cell numbers for downstream functional analyses. This system maintains microglial identity, as confirmed by high expression of canonical microglial markers and high culture purity (Fig. 1). Proteomic analysis revealed that *Tpc2*-deficiency led to a preferential reduction of multiple pro-angiogenic and immune-modulatory factors in retinal microglia, particularly in the secretome (Fig. 2). For instance, TGF-β1 plays a context-dependent role in angiogenesis, where it promotes endothelial cell migration, extracellular matrix deposition, and vessel stabilization during early neovascular responses. In ocular microenvironment, TGF-β signaling has been implicated in choroidal neovascularization and fibrotic remodeling associated with nAMD [38]. HDGF, originally identified as a mitogenic factor for endothelial cells, has been shown to enhance endothelial proliferation, migration, and survival, and its upregulation has been reported in multiple angiogenic and inflammatory settings [39]. In addition, CHIL3 exhibited one of the most pronounced decreases, it is a chitinase-like protein (CLP) belonging to the GH18 chitinase family, a group of secreted glycoproteins involved in extracellular matrix remodeling and immune regulation [40–42]. Notably, it was found that the human functional paralog of CHIL3, CHI3L1 (YKL-40), is elevated in the serum of patients with neovascular AMD [43], which supports the translational relevance of our mouse findings and suggests that TPC2-dependent regulation of CLP secretion may function analogously in human diseases.

Mechanistically, the reduction in pro-angiogenic factors observed in *Tpc2*-deficient microglia is likely mediated by multiple convergent and non-mutually exclusive processes. At the transcriptional level, TPC2 is associated with pathways involving the MiT/TFE family, including transcription factor EB (TFEB) and microphthalmia-associated transcription factor (MITF). They coordinate lysosome biogenesis, autophagy, and cellular energy homeostasis in response to stress and nutrient status [44, 45]. For example, lysosomal Ca²⁺-dependent signaling pathways, exemplified by the CD38–LRRK2 axis, can activate TFEB, suggesting that lysosomal ion channels such as TPC2 may indirectly contribute to TFEB regulation [46]. In melanoma, TPC2 has been shown to regulate intracellular signaling pathways involving Rab7a and β-catenin, ultimately modulating MITF activity and downstream transcriptional programe in migration, invasion, proliferation and tumor growth [47]. These transcriptional programs ultimately link lysosomal status to inflammatory and metabolic adaptations, providing a conceptual hypothesis for how TPC2-dependent lysosomal signaling may influence cell phenotype.

At the level of vesicle trafficking and secretion, TPC2-dependent endolysosomal Ca²⁺ signaling may influence extracellular protein abundance through different mechanisms. TPC2-mediated Ca²⁺ release regulates the motility of endolysosomal vesicles and intracellular cargo transport, thereby facilitating the delivery of secretory cargo [48, 49]. In addition, TPC2 has been shown to control lysosomal positioning, and its loss can result in perinuclear clustering of acidic organelles, potentially limiting their participation in secretory pathways [50]. Local Ca²⁺ microdomains generated by TPC2 also contribute to membrane fusion events required for vesicle maturation and cargo trafficking [51, 52]. TPC2-dependent lysosomal Ca²⁺ signaling has been also implicated in lysosomal exocytosis and other regulated secretory processes [16], suggesting that impaired protein release may contribute to the reduced extracellular abundance of pro-angiogenic factors observed in *Tpc2*-deficient microglia. Consistent with this model, our proteomic analysis revealed a marked reduction of LAMP1 and Rab35 in the *Tpc2*^⁻/⁻^ retinal microglia secretome (Fig. 2C), supporting a defect in vesicle-mediated secretion and endolysosomal trafficking in the absence of TPC2 [53, 54]. Finally, altered endolysosomal maturation and cargo sorting may redirect proteins from secretory pathways toward intracellular degradation, further reshaping the extracellular proteome [55]. In addition, the autocrine/paracrine signaling loop may be altered in the absence of TPC2. For example, *Tpc2* deficiency may reduce the secretion of cytokines such as IL-4, IL-13, and IL-33 from other retinal cell types. These cytokines are known to stimulate retinal microglia to produce pro-angiogenic factors, including CHIL3 [56–59]. Together, these non-mutually exclusive mechanisms are likely to act in concert to produce the observed phenotype.

By using functional assays in which CHIL3 was applied as a representative pro-angiogenic stimulus, we observed that angiogenesis-associated signaling responses were attenuated in *Tpc2*-deficient vascular cells (Fig. 3). This finding aligns with studies, which demonstrated that *Tpc2* KO alters metabolism and protein translation, leading to reduced ERK1/2 expression and impaired MAPK signaling [60]. While pro-angiogenic extracellular proteins are known to activate EGFR-MAPK signaling cascades in multiple cellular contexts, thereby coupling paracrine signals to proliferative and angiogenic responses [61–63], paracrine molecules derived from retinal microglia are insufficient to elicit robust angiogenic signaling in the absence of TPC2. What’s more, conditioned medium from WT microglia robustly induced *Il6* mRNA expression in choroidal vascular cells, whereas conditioned medium from *Tpc2*^⁻/⁻^ microglia failed to elicit a comparable response (Fig. S3). The induction of *Il6* in WT vascular cells is likely mediated by MAPK/ERK and PI3K–NF-κB signaling cascades and promotes *Il6* transcription through AP-1 and NF-κB [64]. Beyond cytokine induction, WT microglia-conditioned medium also promoted the upregulation of lysosomal enzymes (LIPA, HEXB, CTSB, CTSC, CTSD, and NAGA) and extracellular matrix–associated proteins (EGFR, ITGB1, ITGAV, ROCK1, FYN, and THBS1) in choroidal vascular cells. The increased expression of lysosomal enzymes is indicative of coordinated activation of EGFR–MAPK and TFEB-dependent programs that regulate lysosomal biogenesis and cathepsin-mediated proteolytic activity [65]. Concurrently, the induction of ECM-associated proteins implicates integrin–FAK–ROCK signaling and its cross-talk with EGFR–MAPK pathways, thereby promoting extracellular matrix remodeling and cytoskeletal organization required for angiogenic sprouting [66]. In addition, total NF-κB and MAPK levels were also reduced in *Tpc2*^⁻/⁻^ choroidal vascular cells compared with WT. As discussed above, *Tpc2*-deficiency can alter metabolic and translational programs, leading to reduced protein expression. In parallel, TPC2 has also been implicated in the regulation of autophagy and lysosome-dependent protein turnover, suggesting that altered protein stability may also contribute to the decreased abundance of signaling proteins [47, 67]. However, we acknowledge that these effects are not necessarily specific to defined microglia–vascular signaling axis, but may also reflect broader alterations in lysosome-dependent cellular states that influence signal responsiveness in both cell types. Together, these observations suggest that *Tpc2*-deficient vascular cells exhibit reduced responsiveness to microglia-derived factors, resulting in impaired activation of downstream signaling pathways.

To further delineate the cell-intrinsic consequences of *Tpc2*-deficiency in endothelial cells, we examined choroidal vascular cells isolated using a sprouting assay that enriches for Emcn⁺/CD31⁺ choroidal endothelial cells (Fig. S1), representing the microvascular endothelium [68]. A particularly striking finding was the marked reduction in the abundance of cathepsin family proteins in the secretome of *Tpc2*^⁻/⁻^ choroidal vascular cells, with CTSD being the most abundant member (Fig. 4). The maturation and secretion of CTSD are critically dependent on lysosomal calcium homeostasis and proper endolysosomal trafficking [16, 67], in which both processes regulated by TPC2. Notably, this defect was accompanied by the loss of Rab15 and Rab32 in *Tpc2*^⁻/⁻^ choroidal vascular cells, two Rab GTPases essential for endosomal trafficking and lysosome-related vesicle exocytosis [69, 70]. Their depletion provides a mechanistic basis for impaired pro-cathepsin transport and Ca²⁺-dependent secretion downstream of *Tpc2* deficiency.

Disruption of cathepsin function is expected to blunt proteolytic ECM remodeling and limit the release of matrix-bound angiogenic factors such as VEGF and FGF, thereby impairing endothelial migration and sprout formation [71]. Several members of the cathepsin family have been implicated in angiogenesis, primarily through extracellular matrix remodeling and modulation of pro-angiogenic signaling [72]. CTSB and CTSL facilitate endothelial cell migration and vessel sprouting by degrading matrix proteins and activating growth factors such as VEGF [73, 74]. CTSS deficiency impairs angiogenesis and tumor growth in a mouse model, supporting a role for CTSS in promoting angiogenesis through modulation of matrix-derived pro-angiogenic factors [75]. Among these, CTSD, a lysosomal aspartyl protease, has been widely implicated in tumor invasion and metastasis, processes that share mechanistic parallels with angiogenic remodeling [76]. Several studies have demonstrated that CTSD promotes ECM degradation and facilitates the release or activation of matrix-bound growth factors. For instance, Garcia et al. showed that CTSD is aberrantly overexpressed and secreted in metastatic breast cancer cells, and ectopic overexpression of CTSD has been shown to increase the malignant phenotype and metastatic potential of tumor cells in vivo, suggesting a pivotal role of this protease in invasive remodeling [77]. Subsequent work by Park et al. (2016) revealed that hTERT upregulates CTSD expression via the early growth response (EGR)−1 pathway, thereby promoting cancer cell invasion [78]. More recently, Zhang et al. demonstrated that CTSD facilitates breast cancer metastasis by driving ubiquitin/proteasome-mediated degradation of hepsin, further underscoring its role in protease network regulation [79]. Although these studies were conducted in cancer models, the molecular mechanisms they describe, such as ECM remodeling, growth factor activation, and protease/protease crosstalk are also fundamental to angiogenesis. Therefore, the observed reduction of CTSD abundance and activity in *Tpc2*-deficient choroidal vascular cells likely contributes to impaired matrix remodeling and angiogenic signaling, providing a mechanistic link between lysosomal dysfunction and defective neovascularization.

From a translational perspective, human iPSCs with *TPC2*^−/−^ were generated and differentiated into induced endothelial cells (iECs) using established protocols [80] (Fig. S6). *TPC2*^−/−^ exhibited significantly impaired angiogenic capacity, as evidenced by reduced tube formation, migration, and sprouting in vitro compared to WT controls. Correspondingly, the secretion of CTSD was decreased in *TPC2*^*−/−*^ iECs, consistent with our observations in primary mouse choroidal vascular cells (Fig. 6). Notably, partial reduction of TPC2 expression was sufficient to blunt the angiogenic potential in both cell types, while the successful AAV-mediated knockout of *Tpc2* in pRMG further demonstrated the feasibility of targeting using viral gene delivery approaches in the retina (Fig. 5).Within this framework, TPC2 is associated with the secretion of a group of pro-angiogenic factors and proteases that depend on intact lysosomal trafficking and exocytosis, highlighting its potential as a therapeutic target to modulate multiple angiogenic drivers simultaneously.

A notable limitation of the present study is that our mechanistic analyses were primarily performed in ex vivo and in vitro systems, which do not fully recapitulate the complexity of choroidal neovascularization in vivo. Although our previous CNV-related observations provide supportive contextual evidence [17], a more detailed characterization of the phenotypic changes in vivo will help us better understand the role of TPC2 in disease progression. It should also be noted that TPC2 is broadly expressed across myeloid lineages [81, 82], and due to technical limitations our study does not fully distinguish between microglia-intrinsic and general myeloid mechanisms. Future studies using conditional knockout models or comparative myeloid analyses will be, thus, required to fully address cell-type specificity. In addition, the use of long-term retinal microglia cultures may introduce gradual phenotypic adaptation despite verification of key microglial markers, and future work using in vivo systems will be important to confirm the phenotypes retinal microglial under physiological and pathological conditions.

## Conclusion

**Fig. 7:**
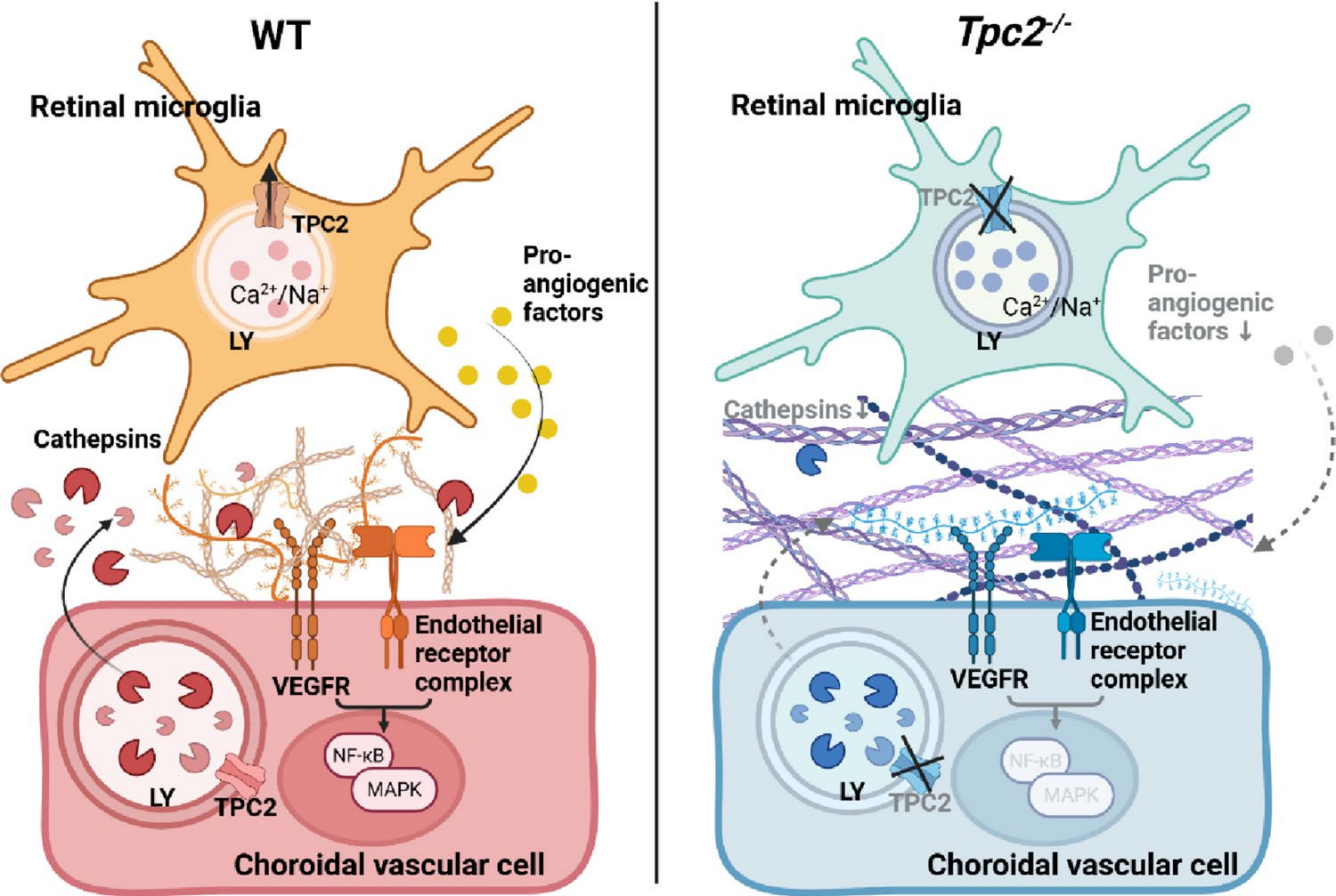
Schematic model of TPC2-mediated microglia-endothelial signaling in choroidal angiogenesis

Our results provide a mechanistic insight, which identifies TPC2 as an important regulator of microglia-endothelial signaling in choroidal angiogenesis (Fig. 7). Loss of TPC2 reduces microglial secretion of pro-angiogenic factors, which in turn attenuates NF-κB and MAPK signaling responses in choroidal endothelial cells, thereby impairing neovascularization. Concurrently, TPC2 modulates endothelial lysosomal function, and secretion of proteases, potentially affecting ECM remodeling. Together, this study suggests that TPC2 represents a potential therapeutic target in nAMD.

In WT conditions, retinal microglia actively secrete pro-angiogenic factors that stimulate choroidal vascular cells, promoting activation of the NF-κB/MAPK signaling pathways, which supports their proliferation and migration by binding endothelial receptor complex. Additionally, choroidal vascular cells in WT mice exhibit normal secretion of cathepsins from lysosomes (LY), contributing to extracellular matrix remodeling in the choroid. In the absence of TPC2, retinal microglia show reduced secretion of pro-angiogenic factors, leading to lower response of the NF-κB/MAPK signaling pathway in choroidal vascular cells, which in turn decreases their proliferation and migration. Concurrently, *Tpc2*-deficiency reduces lysosomal secretion of cathepsins from choroidal vascular cells, which may impair neovascularization. Illustration was generated with biorender.com.

## Supplementary Information

Below is the link to the electronic supplementary material.

Supplementary Material 1

## Data Availability

Data are available via ProteomeXchange with identifiers PXD081654 and PXD081711.
