# Supplementary material for: Lysosomal channel TPC2 modulates microglia-endothelial signaling in choroidal angiogenesis
Source: Angiogenesis. 2026 Aug 12;29(4):58. doi: 10.1007/s10456-026-10082-4 (PMC13469513; doi:10.1007/s10456-026-10082-4)
Supplement: Supplementary file 1 [file 10456_2026_10082_MOESM1_ESM.docx]

**Fig. S1 TPC2 is expressed in retinal microglia and choroidal vascular cells**

**A.** Schematic workflow illustrating the isolation and culture of primary choroidal vascular cells. **B.** Flow cytometric analysis of choroidal vascular cells, showing co-expression of Endomucin and CD31 in endothelial cells. **C.** Relative mRNA expression levels of *Tpc2* in the retina and RPE/choroid of mice at different ages. **D.** Immunofluorescence staining of TPC2 in Endomucin-positive choroidal endothelial cells. Data are shown as mean ± SEM. Illustration in panel (A) was generated with biorender.com.

**Fig. S2 NF-κB and MAPK signaling responses in choroidal vascular cells**

**(A-C)** Quantification of bands intensity of NF-κB (A), P38 MAPK (B), ERK1/2 (C) and their phosphorylated forms in choroidal vascular cells from WT and *Tpc2*^⁻/⁻^ mice. Data are shown as mean ± SEM. Two-way ANOVA was used for statistical analysis:

(A) 15min: WT VEGF only vs. WT VEGF+CHIL3, *p*=0.0073; WT VEGF+CHIL3 vs. *Tpc2*^-/-^ VEGF+CHIL3, *p*=0.0136. 30min: WT VEGF only vs. WT VEGF+CHIL3, *p*=0.0455.

(B) 30min: WT VEGF+CHIL3 vs. *Tpc2*^-/-^ VEGF+CHIL3, *p*=0.0103

(C) 0min: WT VEGF only vs. *Tpc2*^-/-^ VEGF only, *p*=0.0103; WT VEGF+CHIL3 vs. *Tpc2*^-/-^ VEGF+CHIL3, *p*=0.0448. 5min: WT VEGF+CHIL3 vs. *Tpc2*^-/-^ VEGF+CHIL3, *p*=0.0402. 5min: WT VEGF+CHIL3 vs. *Tpc2*^-/-^ VEGF+CHIL3, *p*=0.0294. 30min: WT VEGF+CHIL3 vs. *Tpc2*^-/-^ VEGF+CHIL3, *p*=0.0183. 60min: WT VEGF only vs. *Tpc2*^-/-^ VEGF only, *p*=0.0153; WT VEGF+CHIL3 vs. *Tpc2*^-/-^ VEGF only, *p*=0.0243; WT VEGF+CHIL3 vs. *Tpc2*^-/-^ VEGF+CHIL3, *p*=0.0087.

**Fig. S3 Effects of retinal microglia secretome on choroidal vascular cells**

**A.** Experimental workflow: Primary retinal microglia were cultured until confluence and then maintained in serum-free basal medium for 48 hours. The genotype-matched conditioned medium (CM) was collected and applied to choroidal vascular cells for another 48 hours. Cells were then harvested for qPCR and proteomic analysis. **B.** qRT-PCR results showing mRNA expression levels of *Vegf*, *Vegfr*2, *Il6*, *Ccl2*, and *Icam1* in choroidal vascular cells treated with retinal microglia CM compared to untreated controls. **C.** Heatmap illustrating differential protein expression in WT and *Tpc2*^⁻/⁻^ choroidal vascular cells treated with CM from WT or *Tpc2*^⁻/⁻^ retinal microglia, based on proteomic analysis. Two-tail t test was used for statistical analysis. Data are shown as mean ± SEM. (***p* < 0.01; ****p* < 0.001; *****p* < 0.0001). Illustration in panel (A) was generated with biorender.com.

**Fig. S4 Pharmacological modulation of TPC2 regulates choroidal sprouting in *ex vivo* explants**

**A.** Representative images of choroidal sprouting on day 4 and day 6 from WT control, TPC2-A1P (5 μM*)*, and SG094-treated (10 μM) explants. **B.** Quantification of sprouting area on day 4. **C.** Quantification of sprouting area on day 6. One-way ANOVA was used for statistical analysis. Data are presented as mean ± SEM. (**p* < 0.05, ***p* < 0.01).

**Fig. S5 Validation of sgRNAs targeting *Tpc2***

**A.** Sanger sequencing chromatograms showing mixed peaks downstream of the PAM site: gray and blue boxes indicate the sgRNA sequences, black box indicates the PAM, and arrows denote the Cas9 cleavage site, indicative of frameshift mutations. **B.** TIDE analysis showing indel formation efficiency. **C.** T7 endonuclease I (T7EI) assay confirming cleavage at the target loci. **D.** Flow cytometry analysis of msTPC2-GFP fluorescence signal following CRISPR-mediated knockout (n = 5). One-way ANOVA was used for statistical analysis. Data are presented as mean ± SEM. (*****p* < 0.0001).

**Fig. S6 Generation and validation of *TPC2* knockout iPSCs and differentiation into endothelial cells**

**A.** PCR genotyping of human iPSCs after CRISPR/Cas9-mediated *TPC2* knockout. **B.** Sanger sequencing confirming the fragment deletion within the *TPC2* coding region. **C.** Western blot analysis showing loss of TPC2 protein expression in knockout iPSCs; β-actin was used as a loading control. **D.** Schematic illustration of the differentiation protocol from iPSCs to induced endothelial cells (iECs). **E.** Representative bright-field images showing morphological changes during iPSC-to-iEC differentiation. Illustration in panel (D) was generated with biorender.com.
